# Supplementary material for: Completion of Genetic Testing and Incidence of Pathogenic Germline Mutation among Patients with Early-Onset Colorectal Cancer: A Single Institution Analysis
Source: Cancers (Basel). 2023 Jul 11;15(14):3570. doi: 10.3390/cancers15143570 (PMC10377669; doi:10.3390/cancers15143570)
Supplement: Supplementary file 1 [file cancers-15-03570-s001.zip › cancers-2473295-supplementary.pdf]

Supplementary Materials:

	**eoCRC (n=124)**	**aoCRC (n=179)**
**KRAS**		
WT	45 (36)	68 (38)
G12D	8 (6)	15 (8)
G13D	5 (4)	3 (2)
G12V	4 (3)	3 (2)
A146T	4 (3)	1 (1)
G12C	2 (2)	1 (1)
G12A	0 (0)	3 (2)
G12S	0 (0)	2 (1)
T20M	1 (1)	1 (1)
A59T	2 (2)	0 (0)
Q61H	1 (1)	2 (1)
Q61K	0 (0)	1 (1)
K117N	0 (0)	1 (1)
A146P	0 (0)	1 (1)
A146V	0 (0)	1 (1)
Unknown	52 (42)	77 (43)
**NRAS**		
WT	66 (53)	97 (54)
G12D	0 (0)	2 (1)
G13D	1 (1)	0 (0)
G13R	1 (1)	0 (0)
R68I	0 (0)	1 (1)
Unknown	56 (45)	79 (44)

Supplemental Table S1: KRAS and NRAS mutations
Abbreviations: dMMR: deficient mismatch repair, LS: Lynch syndrome, eoCRC: early-onset colorectal cancer, aoCRC: average-onset colorectal cancer
* One had both K117N and T20M

	Completed testing (n=84)	Did not complete testing (n=40)
**Age (mean)**	41.08	40.48
**Sex**		
Male	47 (56)	26 (65)
Female	37 (44)	14 (35)
**Race**		
White, not Hispanic	75 (89)	32 (80)
Black	0 (0)	3 (8)
Hispanic	2 (2)	0 (0)
Asian	2 (2)	1 (3)
Am. Indian/Alaska Native	0 (0)	2 (5)
Other/Unknown	5 (6)	2 (5)
**BMI near diagnosis**		
<25	26 (31)	7 (18)
≥25, <30	17 (20)	14 (35)
≥30	29 (35)	15 (38)
Unknown	12 (14)	4 (10)
**Stage at diagnosis**		
0	2 (2)	4 (10)
1	10 (12)	3 (8)
2	20 (24)	5 (13)
3	30 (36)	14 (35)
4	17 (20)	11 (28)
Unknown	5 (6)	3 (8)
**Personal history of malignancy**		
Yes	20 (24)	8 (20)
No/unknown	64 (76)	32 (80)

Supplemental Table S2: Patients with eoCRC who completed germline testing versus those who did not
*Abbreviations***:** eoCRC: early-onset colorectal cancer, BMI: body mass index

	**eoCRC (n=84)**	**aoCRC (n=109)**
All patients	25 (29.8)	33 (30.3)
MSH3	3 (3.6)	3 (2.8)
*APC***	5 (6.0)	0 (0)
BRCA2	0 (0)	4 (3.7)
ATM	3 (3.6)	1 (0.9)
POLD1	2 (2.4)	2 (1.8)
CHEK2	2 (2.4)	1 (0.9)
MUTYH	1 (1.2)	2 (1.8)
CDH1	1 (1.2)	2 (1.8)
BARD1	1 (1.2)	2 (1.8)
BLM	2 (2.4)	1 (0.9)
MLH3	2 (2.4)	0 (0)
TSC2	0 (0)	2 (1.8)
BMPR1A	1 (1.2)	1 (0.9)
KIT	1 (1.2)	1 (0.9)
POLE	0 (0)	2 (1.8)
CTNNA1	0 (0)	2 (1.8)
MLH1	0 (0)	1 (0.9)
SDHB	1 (1.2)	0 (0)
EGFR	0 (0)	1 (0.9)
BAP1	0 (0)	1 (0.9)
TP53	0 (0)	1 (0.9)
NF1	0 (0)	1 (0.9)
FH	0 (0)	1 (0.9)
NBN	1 (1.2)	0 (0)
RECQL4	0 (0)	1 (0.9)
CEBPA	1 (1.2)	0 (0)
BRIP1	1 (1.2)	0 (0)
AXIN2	0 (0)	1 (0.9)
SMARCA4	0 (0)	1 (0.9)
WRN	0 (0)	1 (0.9)
*HOXB13****	0 (0)	1 (0.9)

Supplemental Table S3: Variants of uncertain significance by age of first CRC diagnosis.

Abbreviations: eoCRC: early-onset colorectal cancer, aoCRC: average-onset colorectal cancer.

* Among patients with eoCRC, 3 had two VUS, 1 had both a likely pathogenic mutation and VUS, 1 had a pathogenic mutation, likely pathogenic mutation, and VUS, and 5 had a pathogenic mutation and VUS. Among patients with aoCRC, 3 had two VUS, 3 had both a pathogenic mutation and variant of uncertain significance. ** One patient had an increased risk allele of APC. *** Increased risk allele for prostate cancer.
